# A High Throughput Screening Assay for Anti-Mycobacterial Small Molecules Based on Adenylate Kinase Release as a Reporter of Cell Lysis

**DOI:** 10.1371/journal.pone.0129234

**Published:** 2015-06-22

**Authors:** Lauren Forbes, Katherine Ebsworth-Mojica, Louis DiDone, Shao-Gang Li, Joel S. Freundlich, Nancy Connell, Paul M. Dunman, Damian J. Krysan

**Affiliations:** 1 Department of Microbiology/Immunology, University of Rochester School of Medicine and Dentistry, Rochester, New York, 14642, United States of America; 2 Department of Pediatrics, University of Rochester School of Medicine and Dentistry, Rochester, New York, 14642, United States of America; 3 Department of Pharmacology, Rutgers University, Newark, New Jersey, 07103, United States of America; 4 Department of Physiology, Rutgers University, Newark, New Jersey, 07103, United States of America; 5 Department of Medicine, Center for Emerging and Re-emerging Pathogens, Rutgers University, Newark, New Jersey, 07103, United States of America; Institut de Pharmacologie et de Biologie Structurale, FRANCE

## Abstract

*Mycobacterium tuberculosis (Mtb)* is well-established to be one of the most important bacterial pathogens for which new antimicrobial therapies are needed. Herein, we describe the development of a high throughput screening assay for the identification of molecules that are bactericidal against Mycobacteria. The assay utilizes the release of the intracellular enzyme adenylate kinase into the culture medium as a reporter of mycobacterial cell death. We demonstrate that the assay is selective for mycobactericidal molecules and detects anti-mycobacterial activity at concentrations below the minimum inhibitory concentration of many molecules. Thus, the AK assay is more sensitive than traditional growth assays. We have validated the AK assay in the HTS setting using the *Mtb* surrogate organism *M*. *smegmatis* and libraries of FDA approved drugs as well as a commercially available Diversity set. The screen of the FDA-approved library demonstrated that the AK assay is able to identify the vast majority of drugs with known mycobactericidal activity. Importantly, our screen of the Diversity set revealed that the increased sensitivity of the AK assay increases the ability of *M*. *smegmatis*-based screens to detect molecules with relatively poor activity against *M*. *smegmatis* but good to excellent activity against *Mtb*.

## Introduction

*Mycobacterium tuberculosis* (*Mtb*) is one of the most important human pathogens. *Mtb* is the causative agent of tuberculosis (TB) and it has been estimated that approximately one-third of the world’s population is infected with this this bacterium [1]. Furthermore, the World Health Organization (WHO) has estimated that as many as 1.5 million people die each year from TB [1]. The vast majority of the cases of TB occur in areas of the world where access to healthcare is poor, resulting in non-existent or sub-standard treatment. Even in resource-rich regions of the world, treatment of TB is very challenging and requires multi-drug regimens and months-long treatment courses [2]. Further complicating this therapy is the emergence of multi-drug resistant TB (MDR-TB) as well as extremely-drug resistant TB (X-TB). In response to this on-going threat to global human health, interest in identifying and developing new agents with activity against *Mtb* has increased and, in 2012, the first new drug for TB in 40 years was approved by the FDA [3]. However, new agents with novel mechanisms of action and improved activity are, and will continue to be, needed in the fight against this pathogen [4].

In general, two approaches to have been taken to identifying anti-TB drugs: target-based and whole cell, phenotypic screens [5]. Although the post-genomic era has made target-based screening feasible, this approach has been very disappointing with respect to anti-infective discovery for reasons that have been well-discussed in the literature [6]. Despite the fact that simple whole cell growth assays are problematic in *Mtb*, a number of the currently used drugs were identified by growth-based screens [7].

The main technical difficulty with growth-based screens with *Mtb* is that its slow growth rate makes traditional high throughput optical density-based screening problematic, although not impossible. Nevertheless, the challenges with traditional growth-based screening have led to the development of alternative assay methods for *Mtb*-based screening as well as the adoption of model organism-based screening. One solution to the problems associated with the slow growth of *Mtb* has been to screen with faster growing *Mycobacterium* species such as *M*. *smegmatis* [8]. Screening with this model organism has a number of advantages including shorter assay time, more convenient biosafety requirements, and high correlation of hit activity between *M*. *smegmatis* and *Mtb*. The primary limitation of screening with *M*. *smegmatis* is that studies directly comparing the hit profiles of attenuated *Mtb* strains with hit profiles of *M*. *smegmatis* have shown that *M*. *smegmatis* screening will miss molecules with activity against *Mtb* and, hence, is less sensitive [8].

Recently, we have developed a cell-death reporter assay for antimicrobial screening that is based on the observation that microbial cells release the intracellular enzyme adenylate kinase into the extracellular growth medium when they lose cellular integrity [9, 10]. The assay was originally developed for antifungal screening [9] and, subsequently, we successfully applied it to Gram-negative and Gram-positive bacteria [10]. Our previous work with these organisms has shown that the AK assay is more sensitive than traditional growth assays. The main reason for this increased sensitivity is that it allows the detection of molecules that cause as little as 10–20% of a cell culture to die without affecting the final stationary phase density of the culture [9]. Such molecules are missed by conventional growth assays because, while their modest activity affects the time a culture takes to reach stationary phase, the final number of cells in the culture is not significantly affected. In principle, these modestly active molecules could be detected by continuous or multiple-time point monitoring of growth but such assays are not practical in the high throughput setting. Thus, we hypothesized that application of the AK assay to *M*. *smegmatis* screening would increase its sensitivity relative to growth or metabolic activity-based assays and provide an increased yield of molecules active against *Mtb*.

In addition to increased sensitivity for molecules active against *Mtb*, the AK assay has additional features relevant to mycobacterial screening. For example, a crucial requirement for any new anti-TB agent is that it possesses bactericidal activity [11]. Indeed, early bactericidal activity has emerged as an important measure of clinical effectiveness of a particular agent. To our knowledge, no previously reported high throughput screening assay of anti-mycobacterial activity is able to directly distinguish between bacteriostatic and bactericidal molecules. Since AK release only occurs when the bacterial cell membrane loses integrity, the assay is specific for bactericidal molecules [9, 10]. As such, an added feature of the AK assay is that it will directly identify mycobactericidal molecules and, thereby, circumvent the need to perform secondary assays to distinguish bacteriostatic hits from the more valuable bactericidal molecules.

Here, we describe the development, validation, and pilot screening for anti-mycobacterial compounds using the AK assay. As detailed below, the AK assay is applicable to the detection of molecules with bactericidal mycobacterial activity, is amenable to high throughput screening with excellent Z’ scores, and identifies molecules at concentrations below their minimum inhibitory concentrations (MIC). We have validated the AK assay in two pilot screens with *M*. *smegmatis*. A screen of a library of FDA-approved drugs confirmed its ability to identify molecules with known anti-mycobacterial activity with a low false negative rate. Finally, a screen of a ChemBridge Diversity set (20,000 molecules) identified a set of hits active against both *M*. *smegmatis* and *Mtb*. In general, *M*. *smegmatis* based screening using traditional growth assays misses many molecules that have activity against *Mtb*, due to the higher intrinsic resistance of the *M*. *smegamatis*. Our data indicate that screening *M*. *smegmatis* with the AK assay increases the likelihood of detecting molecules with low activity against *M*. *smegmatis* but good activity against *Mtb*. Consequently, the logistical advantages of *M*. *smegmatis* screening can be exploited without the decrease in sensitivity associated with growth based assays.

## Materials and Methods

### Bacterial strains and growth conditions

*Mycobacterium smegmatis* mc^2^155 (generously provided by Dr. Martin Pavelka, University of Rochester) was maintained in Middlebrook 7H9 broth (Becton, Dickinson, and Company, Franklin Lakes, NJ) and plated on Middlebrook 7H10 agar (Becton, Dickinson, and Company, Franklin Lakes, NJ), both supplemented with 0.05% Tween-80 (Fisher Scientific BP338-500) and 0.2% Glycerol (Fisher Scientific BP229-1). Broth cultures were grown on a rotary shaker (225 rpm) at 37°C for 48 hrs.

### Chemicals and compound libraries

The library of FDA-approved drugs was obtained from Selleck Chemical (Houston, TX). A selection of 20,000 structurally diverse small molecules from the ChemBridge DIVERset library (ChemBridge Corporation, San Diego, CA) was available to us through the High Throughput Screening Core at the University of Rochester (Rochester, NY). Streptomycin was acquired from Life Technologies (Grand Island, NY). ToxiLight BioAssay kits were acquired from Lonza (Basel, Switzerland).

### Minimum inhibitory concentration (MIC) testing

To determine the MIC of test compounds against *M*. *smegmatis* mc^2^155, overnight cultures were inoculated 1:100 in 25 ml fresh 7H9 media and grown to exponential phase (~1 X 10^8^ CFU/ml). Exponential phase cells were diluted to cell density of 3 X 10^7^ CFU/ml. A 96-well plate (Costar 3788, Corning, Inc., Corning, NY) containing fresh medium and a dilution series of the test compound (0 to 256 μg ml^-1^) was inoculated with 3 X 10^5^ CFU of mc^2^155 (total volume: 100 μl). Plates were incubated at 37°C for 24 hours. The MIC was considered the lowest compound concentration that inhibited bacterial growth, as judged by the unaided eye. For *M*. *tuberculosis* MICs, stocks of each compound were prepared in DMSO (final concentration of 12 mg/ml). Serial dilutions generated test concentrations ranging between 32 μg/ml and 0.244 μg/ml. *M*. *tuberculosis* strain H37Rv at the mid-logarithmic stage of growth (OD_580_ = 0.4) was diluted 1:100, and 0.1 ml added to each well of a 96-well plate along with 0.1 ml test compound solution. After 6 days of incubation at 37^°^C, AlamarBlue (Invitrogen) reagent was added along with 12.5 μl of 20% Tween80 (Sigma) to evaluate bacterial cell viability; plates were scanned 24 hr later at absorbance 570 nm with a reference wavelength of 600 nm. Inoculum control wells of untreated H37Rv were used to create a survival inhibition curve with each assay. Rifampin in DMSO was used as a positive control (1.6–0.00625 μg/ml). The minimum bactericidal concentration for *M*. *smegmatis* (MBC) was determined by plating the contents of wells from MIC experiments at concentrations equal to and above the MIC. The MBC was determined as the concentration that reduced the CFU/mL > 2 log10 relative to initial inoculum.

### Adenylate kinase assay in 96-well plate format

Overnight cultures of *M*. *smegmatis* mc^2^155 were diluted 1:100 into 25 ml fresh media and grown to exponential phase (~1 X 10^8^ CFU ml/mL) on a rotary shaker at 37°C. The cell were harvested by centrifugation (3000 rpm; 10 min). The supernatant was discarded and the resulting cell pellet was washed three times with 2.5 ml of fresh 7H9 media (2.5 mL). The final cell pellet was re-suspended in 2.5 ml media. In a 96-well white-walled plate, 98 μl of cell suspension (1 x 10^5^ CFU/mL) was combined with the antibiotic or test molecule (2 μL of DMSO solution, final DMSO ≤ 1%). Plates were incubated at 37°C for 6 hours and then equilibrated to room temperature for 30 min. AK detection reagent (Lonza ToxiLight, 100 μL) was added to each well and the plate was incubated in the dark for 30 min at room temperature. Luminescence was measured with an integration time of 1000 ms per well on a SpectraMax M5 plate reader. For the Selleck library screen, the final compound concentration in each well was 5 μM.

### Adenylate kinase assay in 384-well format

*M*. *smegmatis* mc^2^155 inoculum was prepared in the same way as described above for the 96-well plate format. In a 384-well white walled plate, 24 μl of a cell suspension (1 X 10^6^ CFU) in 7H10 media (25 μL) was treated with test compound or DMSO control (final DMSO≤ 1%). Plates were incubated for 6 hours at 37°C and then equilibrated to room temperature for 30 min before addition of AK detection reagent (25 μL; Lonza ToxiLight). The plates were incubated in the dark for 30 min at room temperature. Luminescence was measured with an integration time of 1000 ms per well using an Envision Plate Reader. For the ChemDiversity set, the final concentration for each compound was 50 μM.

### Z’-factor determination

*M*. *smegmatis* mc^2^155 inoculum was prepared in the same way as described above for the 96-well plate format. To each well of a 384-well white walled plate (Costar 3570, Corning, Inc., Corning, NY), 1 X 10^6^ CFU and fresh media were added. Alternating columns were treated with H_2_O or streptomycin (final drug concentration: 2 μg/ml) of as positive control. Total well volume was 25 μL. The plate was incubated for 5 1/2 hours at 37°C. The plate was equilibrated to room temperature for 30 min, treated with AK detection reagent (25 μL) incubated in the dark for 30 min at room temperature. Luminescence was measured at all wavelengths (from 250 nm– 850 nm) with an integration time of 1000 ms per well on a SpectraMax M5 plate reader. Z’-factor scores as previously described [12].

## Results

### Adenylate kinase release is a reporter of mycobactericidal activity of small molecules

The rationale for adenylate kinase (AK) release as a reporter of mycobacterial cell death is based on the fact that AK is exclusively intracellular. When cell integrity is lost, AK is released in to the extracellular space. The activity of the released AK is then measured through a coupled reaction. AK converts two molecules of ADP to ATP and AMP. The ATP generated by this reaction drives luciferase, leading to a luminescent readout of cell lysis. The reagents are commercially available and are compatible with a simple, single plate “add and read” assay platform, ideal for high throughput screening.

Initial pilot studies indicated that streptomycin, an antibiotic with mycobactericidal activity, induced AK release in *M*. *smegmatis* strain mc^2^155 relative to mock-treated control. We then optimized culture inoculum and volume, incubation time, reaction volume, and AK assay time to maximize signal and reproducibility (data not shown). Based on these studies, we found that exponential phase cells were optimal and wells containing cells at a density of 1 x 10^6^ CFU/mL provided the maximum sensitivity. In addition, drug exposure times of 6 h provided good sensitivity; prolonged drug exposure times lead to decreased signal, possibly due to degradation of the AK enzyme in the presence of dead cells (data not shown). Lastly, maximal signal was obtained when an AK reaction time of one hour was used; beyond that time, the signal is no longer linear with time (data not shown). As with these previous protocols, it is crucial to cool the plate to ambient temperature to avoid edge effects due to the temperature dependence of the AK reaction. These conditions are very similar to those previously optimized for fungi and Gram positive/negative bacteria.

**Fig 1:**
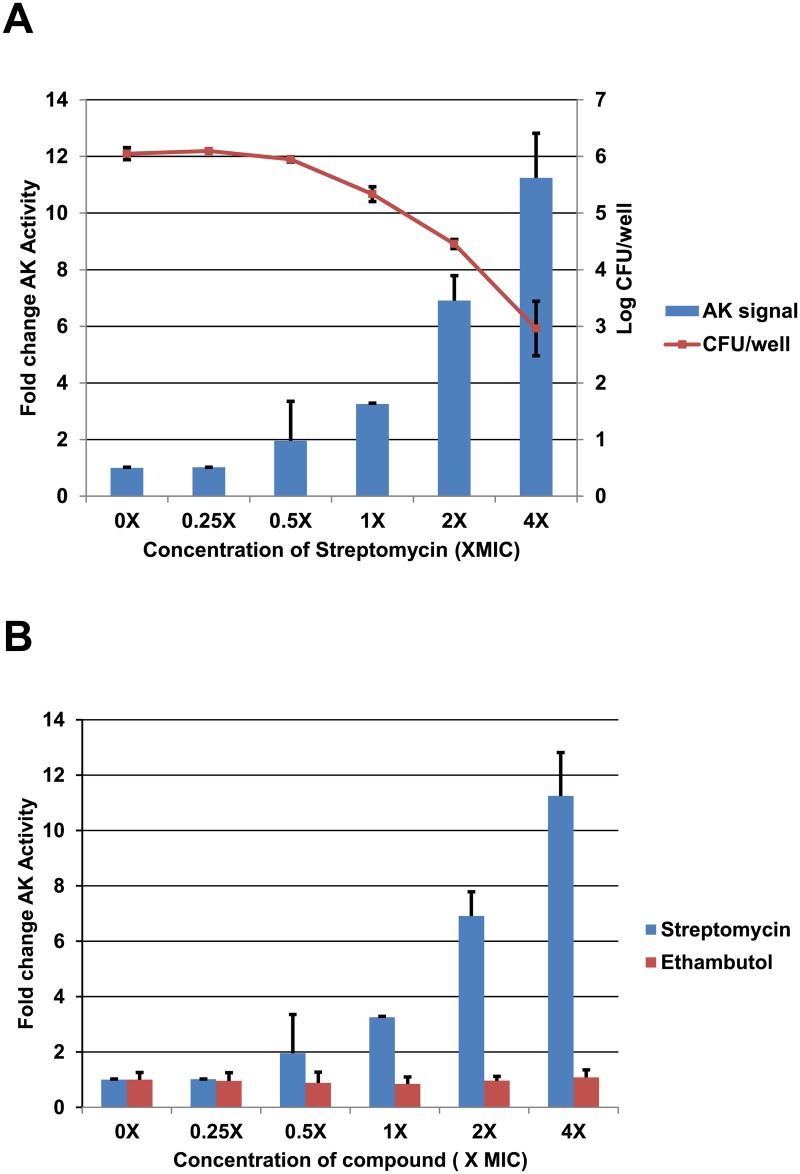
Bactericidal but not bacteriostatic drugs induce adenylate kinase release from *M*. *smegmatis*. A. Exponential phase *M*. *smegmatis* was treated with a dilution series of streptomycin (MIC 0.5 μg/mL). The adenylate kinase activity of the supernatants and the number of viable cells for each concentration were determined after 6 hours of exposure as described in Materials and Methods. B. The adenylate kinase release induced by streptomycin is compared to that with the bacteriostatic drug ethambutol.

Fig 1A shows a dose response for AK release induced by a streptomycin dilution series (0.125 μg/mL to 2 μg/mL) that ranges from 4-fold below to 4-fold above the MIC of streptomycin toward mc^2^155 (0.5 μg/mL). A smooth dose-response curve relating streptomycin concentration to AK activity in the culture medium was generated. Cell viability (CFU/mL) at each streptomycin concentration was also determined by plating the contents of the well. For these optimized conditions, we observed a two-fold increase in AK release relative to untreated cultures at 0.5XMIC, a concentration where there was no detectable decrease in culture viability. This suggests that AK release is a more sensitive measure of bactericidal antimicrobial activity than conventional growth-based assays. This high sensitivity is consistent with previously reported data with fungi and other bacterial pathogens [9, 10], indicating that the AK assay can detect as few as 500 lysed cells in a sample of 10^6^ cells and emphasizes the greater sensitivity of the AK assay relative to growth-based assays.

For fungi, Gram positive bacteria, and Gram negative bacteria, extracellular AK activity increases only when the organisms are exposed to fungicidal/bactericidal agents and no increase is observed with bacteriostatic agents [9, 10]. To confirm that the AK assay was also specific for bactericidal agents in Mycobacteria, we measured the AK release induced by exposure *of M*. *smegmatis* to the bacteriostatic drug ethambutol (Fig 1B). Consistent with expectations, ethambutol at four-fold above MIC failed to induce AK release indicating that bacteriostatic activity is not detected by the AK assay.

**Fig 2:**
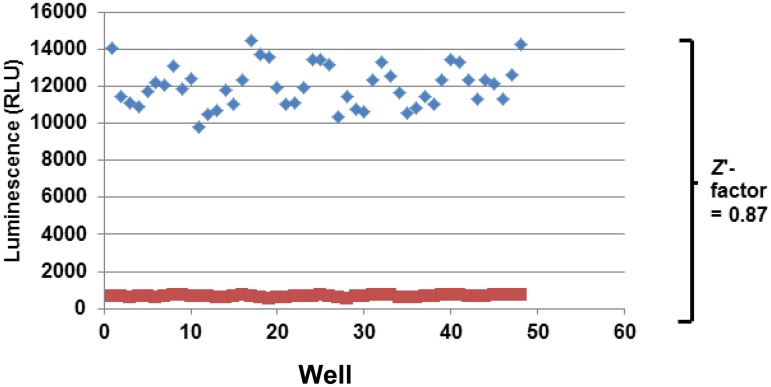
Adenylate kinase release from *M*. *smegmatis* is compatible with high throughput screening. Scatter plot of raw data from an experiment in which alternating rows of a 96-well plate contained either streptomycin (2 μg/mL; 4X MIC) or carrier (H_2_O). The wells were processed as described in materials and methods. The Z’ factor was determined as described by Zhang et al. [12].

In order to further validate the mycobacterial AK assay as an HTS platform, we determined the Z’ score for the assay in 96-well format with alternating columns of DMSO negative control (1%) and streptomycin (2 μg/mL) using the conditions described in the materials and methods. A scatter plot of the raw data from a representative screening plate is shown in Fig 2. The apparent sinusoidal variation is due to a small edge effect. The *Z*’ score for the depicted experiment was 0.87 and is well above the standard 0.5 cut-off for an HTS compatible assay [12]. Similar results were obtained in 384-well format with a Z’ of 0.76 (data not shown). Thus, the AK assay is an HTS compatible assay for the identification of molecules with bactericidal activity towards *M*. *smegmatis*.

### Pilot screen of *M*. *smegmatis* using a library of FDA-approved/off-patent drug-like molecules.

**Fig 3:**
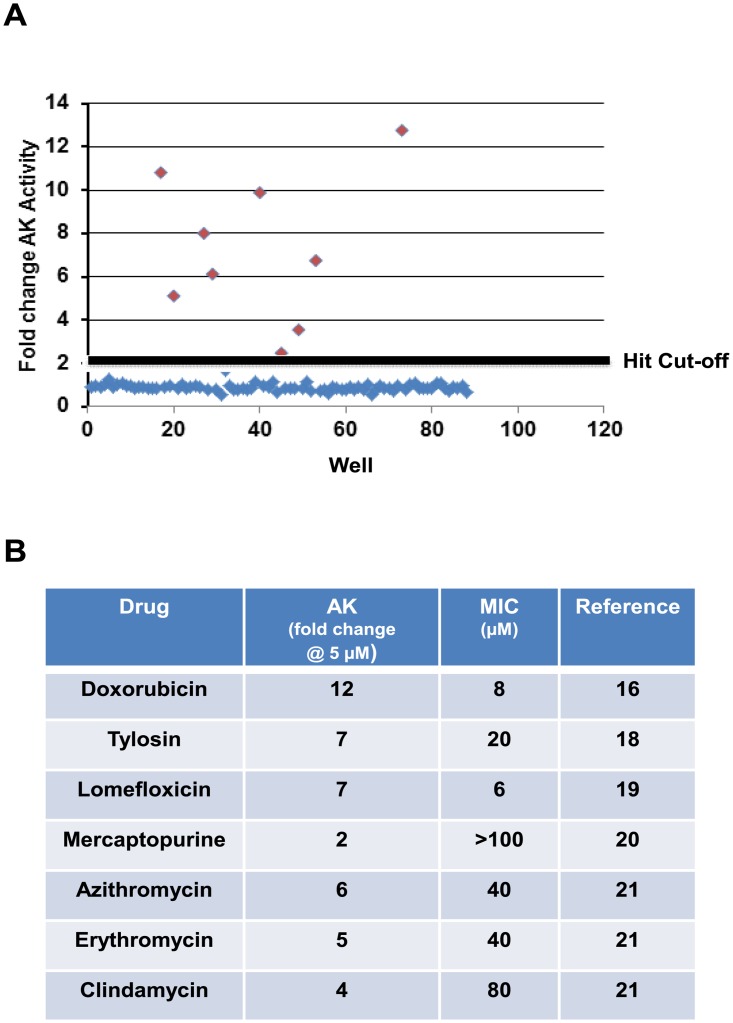
Screen of library of FDA-approved library validates the adenylate kinase assay and indicates it is more sensitive than growth-based assays. A. Scatter plot of raw data from primary screen of the Selleck FDA-approved drug library. The cut-off for hit identification (2-fold increase in AK activity) is indicated by the solid line. B. Table of representative hits from screen that have MIC values above the concentrations at which the screen was performed, indicating that the adenylate kinase assay is more sensitive than growth assays.

To evaluate the performance of the AK assay for HTS with *M*. *smegmatis*, we initially screened the Selleck library of 1163 FDA-approved/off-patent drugs. The library was selected for this pilot screening because it contains a large number of antibacterial agents with well-established anti-mycobacterial activity including fluoroquinolones, rifampin derivatives, streptomycin and other aminoglycosides [13]. As such, the library allowed us to assess both the sensitivity and specificity of the AK assay in the HTS setting. We carried out the screen in 96-well format using the procedure described above for the Z’-score determination. The final concentration of the drugs was 5 μM with <1% DMSO per well. Positive (streptomycin at 2X MIC) and negative control wells (DMSO only) were processed during the screen and gave the expected signal-to-noise (7.4 fold increase in AK relative to untreated). The cut-off for determination of a hit was a 2-fold increase in AK signal relative to DMSO-only wells and a total of 62 hits were identified in the primary screen (hit rate ~5.3%). An example of a representative plate from the screen is shown in Fig 3A. As observed with smaller scale assays, the background was uniformly low across the plates and hits were readily identified. All 62 hits identified in the primary screen were re-tested at the screening concentration of 5 μM and 55/62 (89%) were confirmed to induce >2-fold increase in AK release. There was no identifiable pattern to the false positives expect all showed less than a 2.5-fold increase in AK.

The full set of confirmed hits is shown in S1 Table. Importantly, streptomycin, the positive control for the assay, was identified while the bacteriostatic ethambutol was not positive in the screen. This observation confirmed that the AK preferentially identifies molecules with bactericidal activity. As expected, the set of hits included other aminoglycosides (e.g., amikacin, tobramycin), rifampin derivatives (e.g., rifabutin), macrolides (e.g., clarithromycin), and fluoroquinolones (e.g., ofloxacin). The screen identified drugs from classes with known anti-mycobacterial activity. Specifically, the library contained 4 rifampin derivatives (rifampin, rifabutin, rifapentine, and rifamixin) and all four were hits. Similarly, all of the fluoroquinolones [13], macrolides [5], and aminoglycosides [4] in the library were identified by the AK assay. Three anti-mycobacterial drugs are notable by their absence from the list of hits: INH, ethionamide, and pyrazinamide. All three of these drugs are bactericidal toward *M*. *smegmatis* and, initially, it was surprising that these were not identified. We suspect that their lack of activity in the AK assay is due to the requirement that these drugs be converted within the mycobacterial cell from a prodrug to the active species [14, 15]. Consistent with that explanation, recent single cell, microfluidic studies of the killing kinetics of INH and rifampin toward *M*. *smegmatis* indicate that INH-induced lysis begins ~6 hr after drug exposure while rifampin causes significant lysis at 4hr. Thus, the short incubation time of the AK assay is likely the reason that these three drugs were missed.

Interestingly, we identified five anthracycline-class anti-tumor agents including doxorubicin and daunorubicin as agents that induced AK release. We also determined that the MIC of doxorubicin against *M*. *smegmatis* was 8 μg/mL and confirmed that it was bactericidal at the same concentrations by time kill experiments (>2 log_10_ reduction in initial inoculum). During the preparation of this manuscript, Gajadeera et al. reported the anti-mycobacterial activity of doxorubicin, daunorubicin and idarubicin [16]. Consequently, our results are consistent with this class of molecules having anti-mycobacterial activity and indicate that that the anthracycline class is mycobactericidal. Three other anti-cancer agents were also identified: 6-mercapto-purine, 5-fluoro-uracil and teniposide. Two of these agents, 5-fluoro-uracil and teniposide, were previously identified in a high throughput screen for anti-TB agents [7]. The activity of 6-mercaptopurine against *Mtb* has been recently reported [17].

Importantly, 7 of the hits (Fig 3B) are drugs with MICs against *M*. *smegmatis* mc^2^155 that are higher than the 5 μM screening concentration [16, 18, 19, 20, 21]. This provides intra-screening validation of the dose response data indicating that AK-based screening is more sensitive than growth assays. As noted above one of the main limitations of screening *M*. *smegmatis* as a surrogate organism for the identification of molecules with activity against *Mtb* is that, with growth based assays [8, 22], *M*. *smegmatis* has reduced susceptibility to many molecules with good activity against *Mtb*. The improved sensitivity of the AK assay will increase the likelihood that molecules with modest activity against *M*. *smegmatis* and good activity against *Mtb* are identified from screens of *M*. *smegmatis*. Taken together, these data indicate that the *M*. *smegmatis* AK assay performs well in the HTS setting and provides a sensitive, specific assay for molecules with bactericidal activity against *M*. *smegmatis*.

### Screen of *M*. *smegmatis* with a diversity library identifies bactericidal anti-mycobacterial agents.

To explore the utility of the AK assay in the HTS setting further, we screened *M*. *smegmatis* strain mc^2^155 against a commercial diversity library containing ~20,000 molecules (final concentration: 50 μM). The primary AK assay was performed in 384-well plate format as described above and a hit was defined as a molecule that induced AK activity that was a standard deviation above the average for each plate; this criterion was used because it was not operationally practical to manually place negative controls in each screening plate. We then performed four secondary assays: 1) repeat AK assay to confirm the hit; 2) MIC determination using standard methods; 3) minimum bactericidal concentration (MBC); and 4) activity against *Mtb* strain H37Rv.

**Fig 4:**
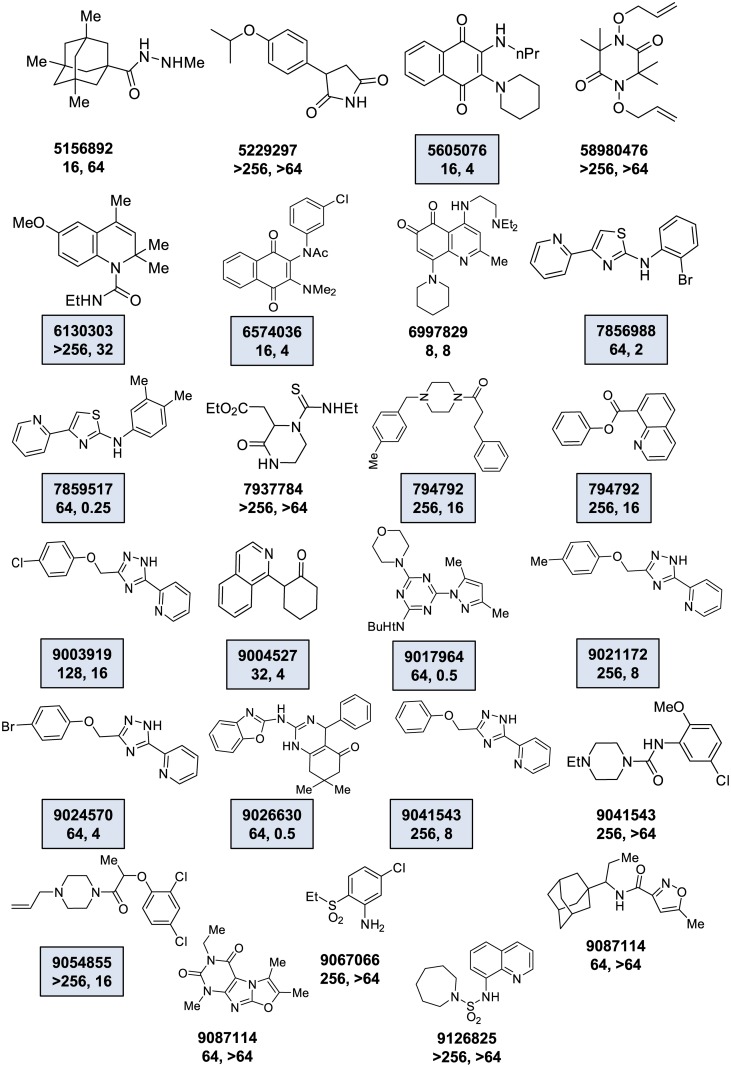
Screen of diversity library with *M*. *smegmatis* identifies molecules with activity against both *M*. *smegmatis* and *M*. *tuberculosis*. The set of molecules identified in a screen of the ChemBridge Diversity Set performed as Materials and methods. The structure of each hit is shown along with the Chembridge ID number. The MIC for the compound against *M*. *smegmatis* is listed first and is followed by the MIC against M. tuberculosis. The boxes highlight molecules which have MIC against *M*. *smegmatis* that is ≥4 folder higher than against *M*. *tuberculosis*.

A set of 25 molecules met the hit criteria which corresponded to a hit rate of 0.125%. All 25 hits were subsequently confirmed to induce increased AK release relative to mock treated controls and their structures are shown in Fig 4. Next, we determined the MIC for each hit against *M*. *smegmatis* using a standard microdilution growth assay. As shown in Fig 4, the molecules that generated increased AK release showed a range of activities by MIC testing (8 to >264 μg/mL).

Next, we characterized the ability of the hit molecules to directly kill *M*. *smegmatis* by determining the MBC for each molecule with an MIC<64 μg/mL. Overall, 6/10 molecules (Fig 4: 6574036; 6997829; 7856988; 7859517; 7952431; 9017964) decreased the initial inoculum by ≥2 log_10_ CFU/mL at concentrations equal to or 4-fold above MIC, indicating that the molecules were bactericidal. Of the 4 molecules (Fig 4: 9024570; 9026630; 9041543: 9087114) that did not reduce inoculum by ≥2 log10, all four had MIC values of 64 μg/mL and the inoculum was reduced by at least 2–4 fold at a concentration of 256 μg/mL, indicating that the minimum bactericidal concentration was greater than 256 μg/mL and that higher concentrations of the molecules are required to achieve the 2 log_10_ reduction.

### Molecules identified in the *M*. *smegmatis*-based AK screen are active against *M*. *tuberculosis*

As discussed above, *M*. *smegmatis* has been used as a convenient model organism for the expedient identification of molecules with anti-mycobacterial activity and, most importantly, activity against *Mtb* [8, 22]. Therefore, we were interested in determining how many of the *M*. *smegmatis* hits identified by the AK assay showed activity against *Mtb in vitro*. We examined all 25 hits by Alamar Blue assay using *Mtb* strain H37Rv. As shown in Fig 4, 18 of the 25 hits were active against *Mtb* at concentrations <64 μg/mL. Interestingly, 15 of the *Mtb*-active hits actually showed lower MIC for *Mtb* than for *M*. *smegmatis*, indicating that the AK assay is more sensitive than growth assays at identifying *Mtb* active drugs by screening the model organism *M*. *smegmatis*.

Of the fifteen ChemBridge library hits with *Mtb* activity, two structural scaffolds were represented by multiple hits: amino-thiazole (Compounds: 7856988 and 7859517, Fig 4) and 2-pyridyl-(1,2,4)-triazole (Compounds: 9003919, 9021172, 9024570, and 9041543, Fig 4). The most active molecule towards *Mtb* was the amino-thiazole derivative 7859517 (MIC 0.25 μg/mL). This scaffold was identified in a previously reported screen [7] and has been subsequently developed further by two groups [23, 24]. Two examples of the pyridyl-triazole scaffold were also identified in the previous screen. The most active pyridyl-triazole from our screen (9024570) was among those previously identified. Re-testing of the molecule with a second lot indicated that the MIC was higher than the original sample (4 μg/mL vs 25 μg/mL) toward *Mtb*. We also confirmed the previous finding that this molecule has significant toxicity against Vero cells (CC_50_ 2–3 μg/mL). Thus, this molecule has intriguing activity against *Mtb* but additional work would be required to address its significant cytotoxicity.

Taken together, the AK assay appears to be a useful approach to improving the sensitivity of *M*. *smegmatis*-based screening for molecules active against *Mtb*. None of these molecules would have been identified by growth assay screening in *M*. *smegmatis* because the MICs toward mc^2^155 were greater than 64 μg/mL for all of the pyridyl-triazoles. Thus, we propose that AK-based screening of *M*. *smegmatis* is an approach to leverage the advantages of this model organism and retain high sensitivity for molecules active against *Mtb*.

## Discussion

High throughput screening for molecules with activity against *Mycobacteria* has a number of technical challenges that make it problematic relative to screening other medically important bacteria. For example, the slow growth of this genus of bacteria means even fast growing species such as *M*. *smegmatis* require a three-day incubation period [8, 22]. This can lead to decreased throughput and also can decrease sensitivity of the screen for relatively unstable molecules. However, recent advances in technologies and innovative uses of reporter strains have improved screening for molecules with activity against *Mycobacteria* [4, 25]. We have described the development and validation of adenylate kinase (AK) release as an assay to rapidly detect molecules that cause loss of cellular integrity in *M*. *smegmatis*. The AK assay has a number of potentially useful features as an approach to high throughput screening for anti-mycobacterial molecules. First, the assay is performed in a simple “add and read” format and requires only a single day of small molecule exposure as compared to the three-day incubation required for standard screening assays such as Alamar Blue-based protocols [7, 8]. Second, we have shown that it is specific for mycobactericidal molecules as demonstrated by the fact ethambutol was negative in both optimization assays and in pilot screens while the vast majority of bactericidal molecules were identified in the pilot screen of the Selleck library. Although this selectivity could be viewed as a liability, the ability of a drug regimen to sterilize sputum cultures is an important predictor of efficacy and bacteriostatic molecules are much less likely to achieve sterilization.

Third, the assay is very sensitive and is able to detect molecules with anti-mycobacterial activity at concentrations well below MIC. For example, a number of the molecules identified in our screen of a ChemBridge Diversity set have MIC values of 256 μg/mL and higher which are at least two-fold higher than the concentration at which we screened (50 μM). The reason for this improved sensitivity is that molecules may cause significant cell lysis (20–30% of a cell population) without causing an effect on the final cell density of an endpoint assay. Although the molecules detected may not be highly desirable per se, they can provide initial hits that could be further developed. Additionally, lower activity hits that share a common structural scaffold with other more active hits can provide useful “intra-screen” structure-activity data. Finally, high sensitivity is particularly important for screening natural product collections where the concentrations of the active components is highly variable and the structure responsible for the activity may be present at sub-inhibitory concentrations.

Although the limitations of screening with the non-pathogenic model organism have been discussed extensively [8, 22], *M*. *smegmatis* screening has led to the identification of useful molecules [8]. Indeed, the most recent addition to the clinical pharmacopeia of anti-mycobacterial drugs, bedaquiline, was identified through a screen of *M*. *smegmatis*. Accordingly, the utility of *M*. *smegmatis*-based screens for anti-mycobacterial drug discovery remains well-recognized. An important limitation of *M*. *smegmatis* as a surrogate organism for *Mtb* drug discovery is sensitivity rather than specificity. As Altaf et al. have demonstrated [8], molecules with activity against *M*. *smegmatis* are usually also active against *Mtb*; however, a significant number of molecules identified in Alamar Blue-based screens of *Mtb* did not have activity against *M*. *smegmatis* and, thus, would have been missed.

Our data indicates that the improved sensitivity of the AK assay can identify some classes of molecules at concentrations below MIC and, as a result, could improve the sensitivity of *M*. *smegmatis*-based screens for some types of molecules that have activity against *Mtb*. For example, the drugs listed in Fig 3 all have MICs that are higher than the concentration at which we carried out the AK assay. By definition, these molecules would have been missed by a growth-based assay. Further supporting this feature of the AK assay is the fact that our screen of the ChemBridge library identified 15 molecules that actually had lower MICs against *Mtb* than they did against *M*. *smegmatis*.

As with any screening assay or technology, AK-based screening of *M*. *smegmatis* has limitations. First, some molecules may require prolonged incubation before they become cidal and, thus, be false negatives. The incubation time for drug exposure described for our standard protocol was chosen to maximize throughput and could be prolonged. However, incubation times beyond 24 hours lead to reduced signal for rapidly cidal drugs, presumably because the adenylate kinase enzyme is not indefinitely stable under the conditions. This relatively short endpoint for the molecule exposures means that some cidal drugs may be missed if the onset of cell lysis is longer than 6 hours. An example of this limitation is the fact that INH was negative in the AK screen, despite its well-known cidal effect on Mycobacteria [26]. Using microfluidic techniques, Wakamoto et al. have shown *M*. *smegmatis* cells do not begin to undergo lysis until after 6 hours of exposure to lethal concentrations of INH. Thus, INH was missed in our screen using the AK assay the drug had not yet begun to cause cell lysis. This is a relatively special case but is a limitation of the assay nonetheless.

Second, some molecules may only be cidal at concentrations above that which are achievable under the screening conditions; growth assays may identify these compounds if they are bacteriostatic at low drug concentrations but the AK assay will miss the entirely. However, we note that other molecules such as aminoglycosides (Fig 1A) cause lysis at concentrations below which they cause a significant reduction in growth. Once again, no assay is perfect and each has advantageous and disadvantages. The AK assay can be expected to identify molecules that a standard growth assay may not and vice-versa.

Third, the increased sensitivity of the AK assay relative to growth assays is not likely to be universally true for all cidal molecules. For example, molecules that directly target membrane, cell wall or other cell integrity mechanisms are likely to represent the bulk of the molecules for which the AK is more sensitive than growth assays. Molecules which cause lysis secondary to other effects on the cell may not be positive or, as discussed above, molecules that cause late onset lysis may also be missed. However, we also note that improved sensitivity is not the only advantage of the AK assay and we feel the ability to directly screen for cidal drugs represents a potentially useful application of this approach.

In summary, AK-assay-based screening of *M*. *smegmatis* appears to offer a number of advantages over growth-based or Alamar blue-based screening. First, the incubation period for the small molecules is 6 hours with the AK assay as compared to 96 hr for the Alamar blue assay. Second, the AK assay is specific for bactericidal molecules and, thereby, reduces the need to use secondary assays to distinguish the more valuable bactericidal molecules from the less useful bacteriostatic hits. Third, the AK assay is more sensitive than standard growth-based assays. Consequently, the AK assay increases the likelihood that the screen will identify molecules with modest-to-poor activity against *M*. *smegmatis* but good activity against *Mtb*. Taken together, we feel that the AK assay represents a useful addition to the repertoire of HTS-compatible assays for the identification of new anti-mycobacterial agents.

## Supporting Information

S1 Table Set of confirmed hits identified in AK screen library of off-patent drugs. (XLSX)
